# Identification of pathogens in the invasive hornet *Vespa velutina* and in native Hymenoptera (Apidae, Vespidae) from SW-Europe

**DOI:** 10.1038/s41598-021-90615-7

**Published:** 2021-05-27

**Authors:** Luis B. Gabín-García, Carolina Bartolomé, Carla Guerra-Tort, Sandra V. Rojas-Nossa, José Llovo, Xulio Maside

**Affiliations:** 1 CiMUS P2D2, Universidade de Santiago de Compostela, Av. de Barcelona s/n, 15782 Santiago de Compostela, Galiza Spain; 2 Instituto de Investigacións Sanitarias de Santiago (IDIS), 15706 Santiago de Compostela, Galiza Spain; 3 Department of Ecology and Animal Biology, Faculty of Sciences, University of Vigo, 36310 Vigo, Galiza Spain

**Keywords:** Pathogens, Ecological epidemiology, Molecular ecology, Invasive species

## Abstract

Invasive species contribute to deteriorate the health of ecosystems due to their direct effects on native fauna and the local parasite-host dynamics. We studied the potential impact of the invasive hornet *Vespa velutina* on the European parasite-host system by comparing the patterns of diversity and abundance of pathogens (i.e. Microsporidia: Nosematidae; Euglenozoa: Trypanosomatidae and Apicomplexa: Lipotrophidae) in European *V. velutina* specimens with those in the native European hornet *Vespa crabro,* as well as other common Hymenoptera (genera *Vespula*, *Polistes* and *Bombus*). We show that (i) *V. velutina* harbours most common hymenopteran enteropathogens as well as several new parasitic taxa. (ii) Parasite diversity in *V. velutina* is most similar to that of *V. crabro*. (iii) No unambiguous evidence of pathogen release by *V. velutina* was detected. This evidence together with the extraordinary population densities that *V. velutina* reaches in Europe (around of 100,000 individuals per km^2^ per year), mean that this invasive species could severely alter the native pathogen-host dynamics either by actively contributing to the dispersal of the parasites and/or by directly interacting with them, which could have unexpected long-term harmful consequences on the native entomofauna.

## Introduction

Insect diversity and abundance are declining worldwide^1,2^. This phenomenon has been associated with parallel effects on wild and managed pollinators, and interpreted as evidence for the deteriorating health of ecosystems and an ongoing major extinction event^3–6^. Pollinator loss is known to have serious consequences on a wide variety of species that rely on them either to feed or to reproduce^4,7,8^, thus threatening biodiversity, ecosystem services and crop-production^9,10^. Anthropogenic drivers of insect loss include climate change, habitat loss, agricultural intensification, exposure to pesticides and pathogens, and the introduction of invasive alien species^6,11–14^.

Over two-thousand invasive alien species have stablished in the terrestrial European Union territory, sixty-six of which have been labelled as of Union concern owing to their potential harm to ecosystems (https://ec.europa.eu/environment/nature/invasivealien/). One such species is *Vespa velutina* Lepeletier, 1836 (Hymenoptera: Vespidae), a hornet of East Asian origin which was first detected in France in 2004 and since then it has spread to all neighbouring countries^15,16^. *V. velutina* can reach very high population densities^17,18^ and is a serious threat to the ecosystem^16,19^. Its most obvious impact derives from its intense predatory activity: in Europe *Apis mellifera* (Hymenoptera: Apidae) represents 30–60% of its catches and it has been estimated that hornet hunting pressure can drive the collapse of around 30% of the colonies^20–22^ and these figures can be as high as 80% for small apiaries (X. Maside unpublished). Their protein diet is completed with other important pollinators, mainly flies, social wasps as well as other arthropods^20,23^. In addition, the sudden irruption of a large hornet population in Europe might also interfere with the native host–pathogen dynamics, further threatening the pollinator system. There is mounting evidence for between-species transmission of protozoan pathogens across a wide variety of pollinator species^24–26^, likely mediated through shared fomites^27,28^. Epidemiological and population genetics data suggest that *Nosema ceranae* (Fungi; Microsporidia; Nosematidae), first described in the Asian honey bee *Apis cerana*, has spread across the *A. mellifera* worldwide population^25,29–31^, from where it might have jumped to bumblebees^24^. Also, *Crithidia bombi* (Euglenozoa: Kinetoplastida: Trypanosomatidae) has been horizontally transferred from commercial to wild bumblebees^32,33^. This and other Trypanosomatidae (i.e. *Crithidia mellificae* and *Lotmaria passim*) are increasingly often found in many pollinators^34–36^. These spillover processes have been related to severe losses in the target host populations and are considered a major threat to ecosystem functioning^24,37,38^. Apart from hunting other insects, the foraging behaviour of *V. velutina* also involves frequent visiting of flowers and other parts of the plants in search of nectar and sap as a source of carbohydrates. Thus, the hornets are frequently exposed to most native pollinator pathogens, some of which might benefit from the hornet’s active contribution to their spread during the hornets’ visits to apiaries and plants, and from the presence of a novel and highly abundant host. These factors could prompt significant variations of the population sizes of the pollinators’ pathogens in Europe^39,40^, but also of their pathogenic properties, such as virulence and contagiousness^41^. Furthermore, the possibility that the founder *V. velutina* individuals that initiated the invasion process might have carried over alien pathogens from its original distribution range in SE Asia, cannot be discarded.

To evaluate the extent to which *V. velutina* interacts with the European native pathogens, a detailed knowledge of the parasites hosted by the invading population and the native entomofauna are needed. But so far, to the best of our knowledge, only three reports on *V. velutina* parasites have been published, and refer to isolated cases of infection by fungi (*Beauveria* and *Metarhizium*), a mermithid nematode (*Pheromermis* sp.) and a parasitoid wasp (*Conops vesicularis*) in France^42–44^. Also, several viruses commonly found in other hymenoptera, including the Israeli acute paralysis virus, the aphid lethal paralysis virus, the black queen cell virus and the Sacbrood virus have been detected in *V. velutina*^45–48^. Here, we hypothesize that in Europe *V. velutina* occupies an ecological niche that largely overlaps with that of native hornets (*V. crabro*) and wasps, such that they interact with a similar profile of parasite species. To falsify this hypothesis, we characterized and compared the patterns of diversity and prevalence of common pollinator enteroparasite species of the groups Nosematidae, Trypanosomatidae and Lipotrophidae, in a collection of samples from Galiza (NW- Iberian Peninsula) representing an European population of *V. velutina*, *V. crabro*, wasps (gen. *Vespula* and *Polistes*) and bumblebees (gen. *Bombus*).

## Results

### Nosematidae

Optical microscopy analysis revealed the presence of spores of *Nosema*-like parasites in the guts of specimens from 19 samples: seven *V. velutina*, four *Bombus* spp*.*, three *Polistes* spp., three *Vespula* spp. and two *V. crabro* (Supplementary Figure S1; Table 1). PCR amplification of the *SSU-rRNA* locus yielded products of the expected size in 24 samples: 12 of them were new positives, but it failed to amplify from seven samples where spores had been detected by microscopy (Supplementary Table S1). This indicates that some samples might harbour *Nosema*-like organisms that were not amplified by the primers used in this study. Only PCR-positive samples were considered in what follows. The most common species was detected in twelve samples (nine of *V. velutina* and three of *V. crabro*) and it was nearly identical to other *Nosema thomsoni* sequences available in GenBank, albeit a single nucleotide substitution (Table 1 and Supplementary Table S1). The sequence reads of two of these *V. velutina* samples (labelled 70 and 163; Supplementary Table S1) displayed double peaks precisely at the six nucleotides of divergence between *N. thomsoni* and *Nosema ceranae*. Cloning of these amplicons confirmed the presence of haplotypes of these two species in both samples. The three positive bumblebee samples produced haplotypes that were nearly identical to *Nosema bombi*. Finally, eight samples—four *V. velutina*, two *Vespula* spp, one *Polistes* spp. and one *V. crabro*—produced a distinct haplotype that displayed 7% nucleotide divergence with *N. bombi* and 13% with *Nosema apis* reference sequences.

**Table 1:** Parasite prevalence across hosts. Relative frequencies (in %) and 95% CI.

	*V. velutina* (*N* = 79)	*V. crabro* (*N* = 31)	*Vespula* spp. (*N* = 21)	*Polistes* spp. (N = 11)	*Bombus* spp. (N = 25)
**Nosematidae**					
*N. ceranae*	2.5 (0.0–6.0)	3.2 (0.0–9.4)	0.0	0.0	0.0
*N. bombi*	0.0	0.0	0.0	0.0	12.0 (0.0–24.7)
*N. thompsoni*	11.4 (4.4–18.4)	9.7 (0.0–20.1)	0.0	0.0	0.0
Other spp.	5.1 (0.2–9.9)	3.2 (0.0–9.4)	9.5 (0.0–22.1)	9.1 (0.0–26.1)	0.0
Any spp.	16.5 (8.3–24.6)	16.1 (3.2–29.1)	9.5 (0.0–22.1)	9.1 (0.0–26.1)	12 (0.0–24.7)
**Trypanosomatidae**					
*C. bombi*	17.7 (9.3–26.1)	12.9 (1.1–24.7)	23.8 (5.6–42.0)	18.2 (0.0–40.1)	48.0 (28.4–67.6)
*C. mellificae*	8.9 (2.6–15.1)	3.2 (0.0–9.4)	4.8 (0.0–13.9)	18.2 (0.0–40.1)	8.0 (0.0–18.6)
*C. acanthocephali*	5.1 (0.2–9.9)	0.0	4.8 (0.0–13.9)	0.0	0.0
*L. passim*	3.8 (0.0–8.0)	16.1 (3.2–29.1)	19.0 (2.3–35.8)	9.1 (0.0–26.1)	4.0 (0.0–11.7)
Other spp.	5.1 (0.2–9.9)	0.0 (0.0–0.0)	4.8 (0.0–13.9)	9.1 (0.0–26.1)	0.0
Any spp.	29.1 (19.0–39.1)	22.6 (7.9–37.3)	39.1 (19.2–59.1)	36.4 (7.9–64.8)	52 (32.4–71.6)
**Lipotrophidae**					
*A. bombi*	17.7 (9.3–26.1)	29.0 (13.1–45.0)	42.9 (21.7–64.0)	45.5 (16.1–74.9)	24.0 (7.3–40.7)
**Any spp. (pooled)**	41.8 (30.9–52.6)	41.9 (24.6–59.3)	81.0 (64.2–97.7)	72.7 (46.4–99.0)	68.0 (49.7–86.3)

Any spp. refers to the pooled fraction of samples with at least one parasite species. These values are usually smaller than the sum of the frequencies of each species because some samples harbour more than one parasite.

**Figure 1:**
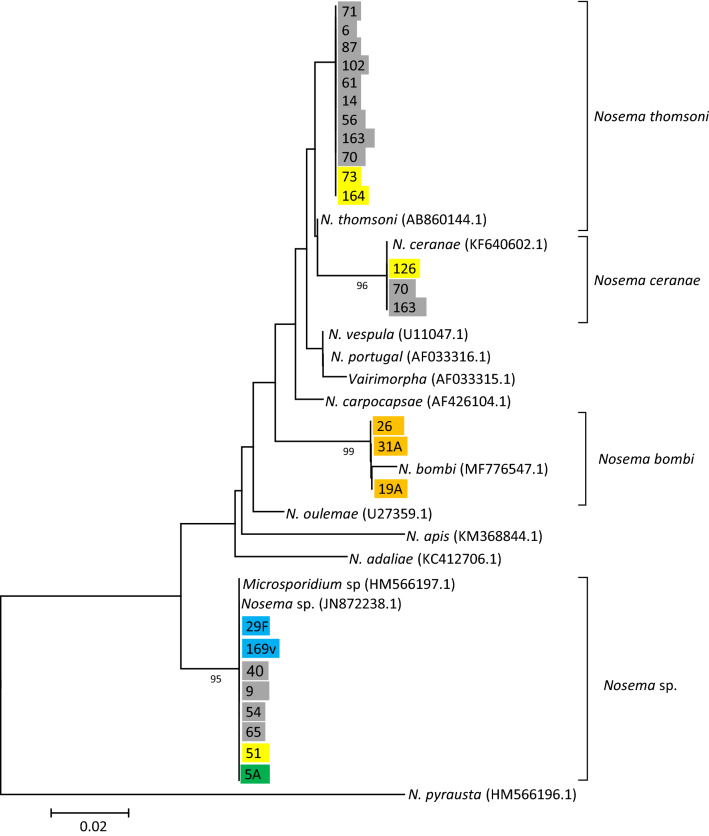
Phylogenetic relationships of the *SSU-rRNA* sequences from Nosematidae. Sequence names indicate the sample name and are color-coded by host groups: *V. velutina* (grey), *V. crabro* (yellow), *Bombus* spp. (orange), *Vespula* spp. (blue), *Polistes* spp. (green). Some sequences that were slightly shorter or with double peaks were excluded. The evolutionary history was inferred using the NJ method. Bootstrap values higher than 70% are shown next to branches. The evolutionary distances were computed using the Tamura 3-parameter method and are in the units of the number of base substitutions per site.

A phylogenetic reconstruction of the evolutionary relationships of the *SSU-rRNA* sequences revealed that most Microsporidia from wasps and bumblebees clustered into two main groups (Fig. 1): the largest one included haplotypes closely related to *Nosema thomsoni* (11), *Nosema ceranae* (3) and *Nosema bombi* (3). The second group, which branched off earlier in the tree, included highly differentiated *Nosema*-like sequences from *V. velutina*, *V. crabro*, *Vespula* spp. and *Polistes* spp. samples, as well as two other sequences that were retrieved from GenBank, one isolated from a bumblebee in China and the other from the European corn borer (*Ostrinia nubilalis¸* Lepidoptera: Crambidae) in France^49,50^. The low diversity, high differentiation and strong bootstrap support for this branch suggests that this clade might correspond to a yet undescribed new microsporidian species present in a wide variety of hymenopteran hosts.

### Trypanosomatidae

PCR amplification of *rpb1* and *topoII* allowed the identification of 56 Trypanosomatidae-positive samples (Table 1 and Supplementary Table S1). The two loci were amplified in twenty-nine of them and just one locus in the remainder: *rpb1* in 13 and *topoII* in 14. In 20 of the former samples the two loci allowed the detection of the same parasite species (Supplementary Table S1), whereas there were discrepancies in the other nine either because one locus allowed the detection of more species than the other (*N* = 5) or because they identified different species (*N* = 4). These differences could not be attributed to a systematic bias of the detection technique—i.e. a differential sensibility of the PCR-primer pairs designed for each locus across parasite species, as both loci allowed the detection of a similar number of positives samples (*N* = 42 and 43, for *rpb1* and *topoII*, respectively) and there were no statistically significant differences between the frequency spectra of the parasites identified by either of them (X^2^ = 7.38; *d.f*. = 4; *P* = 0.11; in a Chi-squared test of homogeneity).

**Figure 2:**
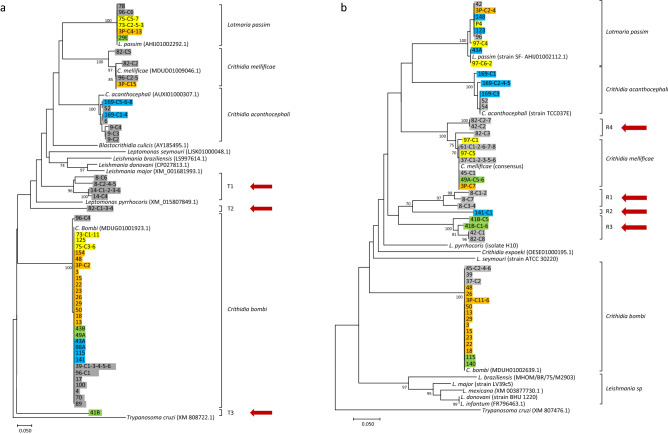
Phylogenetic relationships of the *topoII* (**a**) and *rpb1* (**b**) sequences from Trypanosomatidae. Sequence names include the sample name and the clone number when applicable (C-followed by clone numbers separated by dashes if more than one). Host groups are color-coded: *V. velutina* (grey), *V. crabro* (yellow), *Bombus* spp. (orange), *Vespula* spp. (blue), *Polistes* spp. (green). Red arrows indicate new clades. Some sequences that were slightly shorter or with double peaks were excluded. The evolutionary history was inferred using the NJ method. Bootstrap values higher than 70% are shown next to branches. The evolutionary distances were computed using the modified Nei-Gojobori method (assumed transition/transversion bias = 2) and are in the units of the number of synonymous differences per synonymous site.

Most positive samples presented *topoII* and *rpb1* haplotypes that were nearly identical to Trypanosomatidae species previously found in honeybees and bumblebees (Fig. 2). The average pairwise neutral divergence between those closely related to *Crithidia acanthocephali*, *C. bombi*, *C. mellificae* or to *L. passim* was 1.1% ± 0.72 and 0.5% ± 0.24 for *topoII* and *rpb1*, respectively (± SE; Supplementary Table S2).

Contrastingly, other *topoII* and *rpb1* haplotypes displayed high divergence from any previously reported sequences. At *topoII* these distinct haplotypes formed three clusters: T1, which included four haplotypes from samples 8 and 14; T2, one from sample 82; and T3, one from 41B (Fig. 2a). Similarly, at *rpb1* there were up to four novel clusters (R1-R4). Three of them were detected in the same samples as those of *topoII*: R1, three haplotypes from sample 8, R3 in samples 41B, 42 and 82, and R4, which included sequences from samples 82 and 42 closer to *C. mellificae* (Fig. 2b). Most of these novel clades displayed levels of genetic differentiation of similar magnitude to those observed between the regular species. For instance, the closest relative of any of the new *topoII* lineages was *Blastocrithidia culicis*, which presented a level of synonymous divergence from clade T3 of 34.7% ± 4.84 (Supplementary Tables S3and S4), a value slightly larger than that observed between *B. culicis*, *L. passim* and *C. acanthocephali* (average *K*_S_ = 33.7% ± 0.04). This is also true for most *rpb1* new clades, except R4 which displayed lower divergence from *C. mellificae* (*K*_S_ = 7.0 ± 2.46 Supplementary Table S4). At any rate, this value was nearly ten times larger than the average diversity observed within species (see above).

These results are consistent with the presence of new Trypanosomatidae taxa in the samples. However, to make further inferences, such as to estimate the total number of new taxa by combining the information from the two loci, is not straight forward because: (i) the evolutionary relationships among taxa might vary significantly across loci due to both deterministic (differential patterns of natural selection) and stochastic effects (i.e. mutation and drift). (ii) The resolution of the PCR technique is expected to vary across samples and loci due to differences in template DNA quality across samples and in the ability of primers to bind new sequences. (iii) There is no minimum threshold value of neutral divergence to unambiguously determine whether variation occurs between or within species.

*C. bombi* was the most common Trypanosomatidae in the sample (22.2%). It was the most prevalent parasite in all hosts but in *V. crabro*, and it reached up to 48.0% prevalence in bumblebees (Table 1). *C. mellificae* and *L. passim* were also detected in all hosts, although at lower prevalence (8.6% and 10.4%, respectively). *C. acanthocephali* was found in four *V. velutina* and one *Vespula* spp. samples (5.1% and 4.8%, respectively).

Forty of the positive samples (71.4%) harboured a single Trypanosomatidae species, thirteen (23.2%) had two and three had three (5.4%) (Supplementary Table S1). Co-occurrence of parasites usually involved the three most prevalent species (*C. bombi, C. mellificae* and *L. passim*). A significant excess of mixed samples relative to random expectations was detected between these three species (*P* < 0.05 in pairwise comparisons assuming a hypergeometric distribution; Supplementary Table S5).

### Lipotrophidae

*Apicystis* parasites were detected in 43 samples (25.7%; Table 1 and Supplementary Table S1). Sequences were identical to other *Apicystis bombi* haplotypes previously found in honeybees (e.g. AB738024), except for two sequences that displayed a single nucleotide substitution. *A. bombi* prevalence was largest in *Polistes* spp. (45.5%) and *Vespula* spp. (42.9%) and lowest in *V. velutina* (17.7%).

### Overall between-hosts comparisons

**Figure 3:**
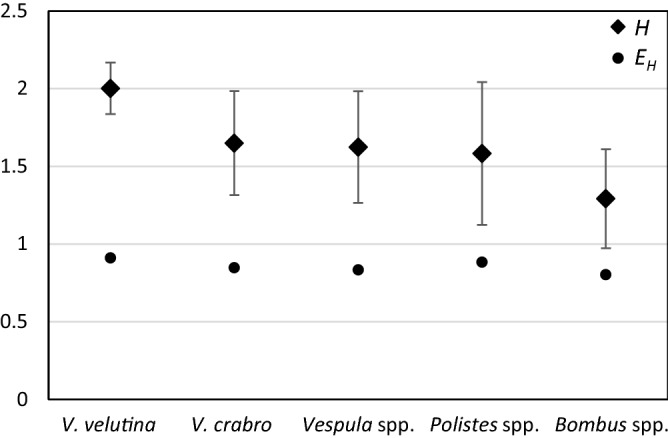
Parasite diversity in the different hosts. Diamonds represent the Shannon diversity Index (*H*; with 95% CIs bars) and dots the Shannon’s equitability index (*E*_*H*_).

Eighty-eight out of the 167 samples tested positive for any of the parasites (52.7%; from data in Supplementary Table S1). The *Vespula* spp. and *Polistes* spp. groups presented the highest fractions of positive samples (81.0% and 72.7%, respectively; Table 1), followed by *Bombus* spp. (68.0%). *V. velutina* and *V. crabro* presented lower frequencies of positive samples (42.0%; Table S1). The two hornet species also presented similar prevalence of parasites of the three groups (X^2^ = 1.22; *d.f*. = 2; *P* = 0.54, in a Chi-square test of homogeneity; data from Table S1). But these figures only represent overall parasite prevalence, and do not consider species variation. For a more comprehensive comparison we used the Shannon diversity and equitability indexes across the five hosts (Fig. 3). *V. velutina* displayed the highest parameter values (*H* = 2.0 and *E*_*H*_ = 0.91) although they were only slightly larger than those for the other three Vespidae hosts. Indeed, there were no significant differences between *H* estimates for *V. velutina* and any of the other Vespidae groups, (*P* > 0.05 in pairwise Student’s *t* tests). Contrastingly, the bumblebees produced the lowest diversity scores, as expected given that they hosted fewer parasite species than any other group (Supplementary Table S1) and that just two of them (*C. bombi* and *A. bombi)* accounted for 75.0% of the positive samples. The differences between *H* for *Bombus* spp. and the Vespidae (pooled data) were statistically significant (t = 5.47; *d.f.* = 31; *P* < 0.001 in a Student’s *t*-test).

The number of parasites species per sample varied significantly across hosts (Table 2). The two hornet species presented very similar distributions (X^2^ = 0.05; *d.f.* = 2; *P* = 0.97, in a chi-square test of homogeneity; samples with 2, 3 or 4 parasites were grouped to avoid zero values), which differed significantly from those of the other three hosts (X^2^ = 19.5; *d.f.* = 2; *P* < 0.001; data from Table 2). Nearly 60% of the hornet samples did not harbour any parasite, about 20% had just one species and a similar fraction had two or more parasites. In contrast, most wasps and bumblebees’ samples were positive (an average of 73.7% across the three hosts), most of them with one parasite (47.4%) and 26.3% with two or more. No significant associations between the three main parasite groups were detected (*P* > 0.05 in all pairwise comparisons, Supplementary Table S5).

**Table 2:** Co-occurrence of parasites in samples across hosts. Number of samples with different number of parasite species. Relative frequencies (%) are given in brackets.

No. of parasite species per sample	*V. velutina*	*V. crabro*	*Vespula* spp.	*Polistes* spp.	*Bombus* spp.
	(*N* = 79)	(*N* = 31)	(*N* = 21)	(*N* = 11)	(*N* = 25)
0	46 (58.2)	18 (58.1)	4 (19.0)	3 (27.3)	8 (32.0)
1	14 (17.7)	6 (19.4)	12 (57.1)	4 (36.4)	11 (44.0)
2	11 (13.9)	3 (9.7)	4 (19.0)	4 (36.4)	5 (20.0)
3	7 (8.9)	4 (12.9)	1 (4.8)	0 (0.0)	1 (4.0)
4	1 (1.3)	0 (0.0)	0 (0.0)	0 (0.0)	0 (0.0)

## Discussion

Microscopy and molecular methods were used to determine the prevalence and diversity of parasites of the groups Nosematidae, Trypanosomatidae and Lipotrophidae in a collection of 167 samples representing the populations of *V. velutina* (79), *V. crabro* (31), *Vespula* spp*.* (21)*, Polistes* spp*.* (11), and *Bombus* spp*.* (25), in Galiza (NW-Iberian Peninsula). Most pathogens commonly found in native European Hymenoptera were detected in the samples. Trypanosomatidae were the most prevalent group of parasites. *C. bombi* was present in up to 48.0% of the bumblebees, a figure slightly larger than previous reports on bumblebees from Scotland^51^, England^27,33^, Argentina^52^ and the Iberian Peninsula^53^. This parasite was also detected in all Vespidae hosts along with *C. mellificae* and *L. passim*; the two latter species at an average frequencies below 10%, in agreement with previous records from honeybees from Galiza^54^ or North America^55^. There was a significant excess of co-occurrence of Trypanosomatidae species in the whole sample. This is consistent with data from honeybees^35,56^. No other associations were detected.

*Apicystis bombi*, a Neogregarine, was the most prevalent parasite of the series (25.7%), reaching frequencies of 40% and greater in *Vespula* spp. and *Polistes* spp. These figures are even greater than those previously reported for this parasite in bumblebees, which are considered as their primary host, elsewhere in Europe and in South America^27,33,51,52^. In the Iberian Peninsula *A. bombi* had been exclusively detected in bumblebees from southern Spain, where *Bombus terrestris* are extensively used in greenhouse tomato production^53^, and its dispersal has been linked to the use of managed *B. terrestris*, as in other parts of the world^33,52^. The present data suggest that this pathogen is widespread across the whole of the Iberian Peninsula and can be found in a wide variety of hosts, including bumblebees, wasps and hornets, as suggested by recent reports of *A. bombi* in honeybees and in wild bumblebees form various locations across Iberia^36,56^.

Nosematidae were the less prevalent of the three parasite groups (14.4%). *N. apis* was not detected, and *N. ceranae* and *N. bombi* were restricted to five samples: two hornet and three bumblebee samples, respectively. This is at odds with recent reports from honeybees and bumblebees, where Nosematids (particularly the latter two species) are usually found at high frequencies in Galiza^54^ and elsewhere^51,57–61^, and with previous evidence that *N. bombi* was circumscribed to bumblebees from the southern Iberian Peninsula^53^. On the other hand, *N. thomsoni*, a species that was first described in the moth *Choristoneura conflictana* (Lepidoptera: Trotricidae) and has been found only sporadically in Apidae, including honeybees, bumblebees and solitary bees^49,56,62,63^, was the most prevalent Nosematidae, although it was detected only in both hornet species.

In addition to the species commonly found in honeybees and bumblebees, the molecular analysis allowed the detection of several new taxa: one microsporidium found in the four Vespidae hosts and five Trypanosomatidae (three in *V. velutina* and two in *Vespula* spp. and *Polistes* spp). *Nosema* sp. had been previously found in a honeybee in China and in the European corn borer (*Ostrinia nubilalis*) in France^49,50^ and one of the Trypanosomatidae in honeybees in the Iberian Peninsula^56^. All these reports postdate the arrival of *V. velutina* in Europe. It could thus be speculated that some of these organisms might have been brought into Europe by the invading *V. velutina*. But the scarcity of available data on pathogen diversity in hymenopterans other than bees and bumblebees from Europe and SE-Asia^64^ hampers the possibility of reliably testing this hypothesis. Provided that the *V. velutina* population in Europe probably derived from a single female^65^, the possibility that it brought along novel parasitic taxa into Europe and that they survived over sixteen generations seems somewhat remote. This goes in line with the lack of evidence for pathogen release by invasive populations of *V. vulgaris* in South America and New Zealand^66,67^. At any rate, the finding of these new parasite taxa highlights that pollinators other than the commonly studied honeybees and bumblebees might harbour a plethora of parasites and that their spread across other pollinator species might have undesirable health consequences for the entire ecosystem.

Three main patterns of parasite presence across the five hosts groups were observed: (i) hornets and wasps presented similar levels of parasite species diversity, somewhat greater than the bumblebees, whose parasite repertoire was dominated by just two species (*C. bombi* and *A. bombi*; Table 1). (ii) Among the Vespidae, the parasite profile of *V. velutina* was most similar to that of *V. crabro*; both displayed a varied representation of the Nosematidae and Trypanosomatidae, whereas the presence of Nosematidae in *Vespula* spp. and *Polistes* spp. was only testimonial and limited to three samples with rare new taxa (Table 1). Also, (iii) most hornet samples were parasite-free, whereas wasps and bumblebee samples often presented one or more parasite species.

The above discordances contrast with the facts that all of them visit flowers in search of food^24,27,28,68^ and that the Vespidae diet includes honeybees and bumblebees, amongst other insects^20,69^. However, subtle variation in their feeding habits might help explain the differences. For instance, (i) Vespidae and Apidae might not have the same flower preferences, such that the spectra of parasite species that they encounter might be different. (ii) The Vespidae feed on nectar, but do not seem to actively harvest pollen while flower visiting, thus they may not frequently ingest the parasites in the flower surface brought about by other pollinators^69,70^. (iii) Wasps often discard the abdomens of their prey, which might reduce their exposure to gut pathogens such as the ones studied here. And (iv) hornets, besides feeding on carrions, use only the thoraxes of their preys and discard all other body parts^20^ (see also: http://www.vespa-crabro.com/) whereas *Vespula* spp. rely more often on dead preys and sporadically include the heads and the abdomens in their diets^69^. Further knowledge of their feeding habits should help ascertain the extent to which they can explain the different patterns of parasite distribution across hosts.

The arrival of *V. velutina* into Europe together with its enormous reproductive success mean that this invasive hornet is a serious threat to the health of the native insect pollinator community. Theoretical approaches identify vast areas of Europe where climatic conditions are now suitable for *V. velutina* reproduction. They comprise most of the Mediterranean and Atlantic coastal areas, including the British Isles^71^, but global warming could facilitate its expansion throughout most continental Europe^72^. In addition, *V. velutina* can reach very high population densities: in Galiza (NW-Iberian Peninsula) the public services removed nearly 70,000 nests in three years (2018 to 2020; data released by Xunta de Galiza). Most of them were in the areas of highest incidence of *V. velutina*, which comprise about 1/5 of the Galician territory (approx. 6000 km^2^; as estimated from Rodríguez-Lado^73^). This means that about four nests were destroyed per km^2^ in these areas. Provided that between 60–70% of the extant nests remain undetected^74^, nest density in Galiza can be approximated as 14–17 nests per km^2^, which fits well with observations of up to 12 nests per km^2^ in Andernos-les-Bains (France)^18^. Considering that each nest on average produce 6150 hornets per reproductive season (June–October)^17^, highly infested areas harbour on average 100,000 V*. velutina* specimens per km^2^ per year. The presence of this huge population is likely to have at least two main effects on the native entomofauna. One is directly derived from the intense predatory activity they exert over a wide variety of insects, including common wasps, paper wasps, bumblebees, honeybees, dipterans (syrphids and brachycera), hymenopterans (halictids) among others^20,23,75^. Indeed, this pressure is so strong that they can destroy nests of *Vespula germanica* (X. Maside unpublished) and drive to extinction up to 30% of the honeybee colonies, on average^21^. These effects are inversely associated with the size of the apiaries due to a difussion effect of the predatory activity on individual colonies. Indeed, smaller-scale professional beekeepers have reported the death of all the colonies in small apiaries (up to 50 colonies) due to *V. velutina* predation^76^.

The second effect has been so far unexplored and refers to the indirect impact of this large novel population on the local host-parasite dynamics. Our data revealed that the invading *V. velutina* harbours most if not all parasites commonly found in native European pollinators. Therefore, its presence is causing a significant change in the host community whose effect will be determined by the specific relationships between *V. velutina* and the parasites, and how its presence modifies the abundance of parasite spreaders, the rate of parasite-host encounters, and the probability of disease transmission^77^. For instance, the presence of a new host might have a dilution effect, particularly for those parasites that find that *V. velutina* is not a competent host, as they will experience a reduction in their chance to find a suitable one^77–79^. Alternatively, it might produce an amplification effect on those pathogens that successfully reproduce in them^80^. Furthermore, a surplus of a competent hosts might prompt selection for virulence^41,81^, threatening the overall health of native pollinators. *V. velutina* might further contribute to pathogen evolution by favouring the encounter and sexual reproduction between strains of pathogens usually found in different hosts on which they prey. The potential role of recombination and natural selection on pathogen evolution is well illustrated by the recent spread of a novel hypervirulent strain of the fungus *Batrachochytrium dendrobatidis* that threatens amphibian diversity worldwide^82^.

Understanding the impact of invasive species in biodiversity is critical to properly protect native ecosystems. Here we showed that specimens of the invasive *V. velutina* in Europe harbour a wide variety of parasites commonly found in local pollinators, but have not investigated the extent to which this interaction alters local host–pathogen dynamics. Further studies are needed to quantify and comprehend the potential consequences of this disturbance and to help put forward strategies to minimize its impact and to protect the already badly threatened native insect populations.

## Methods

### Sampling

**Figure 4:**
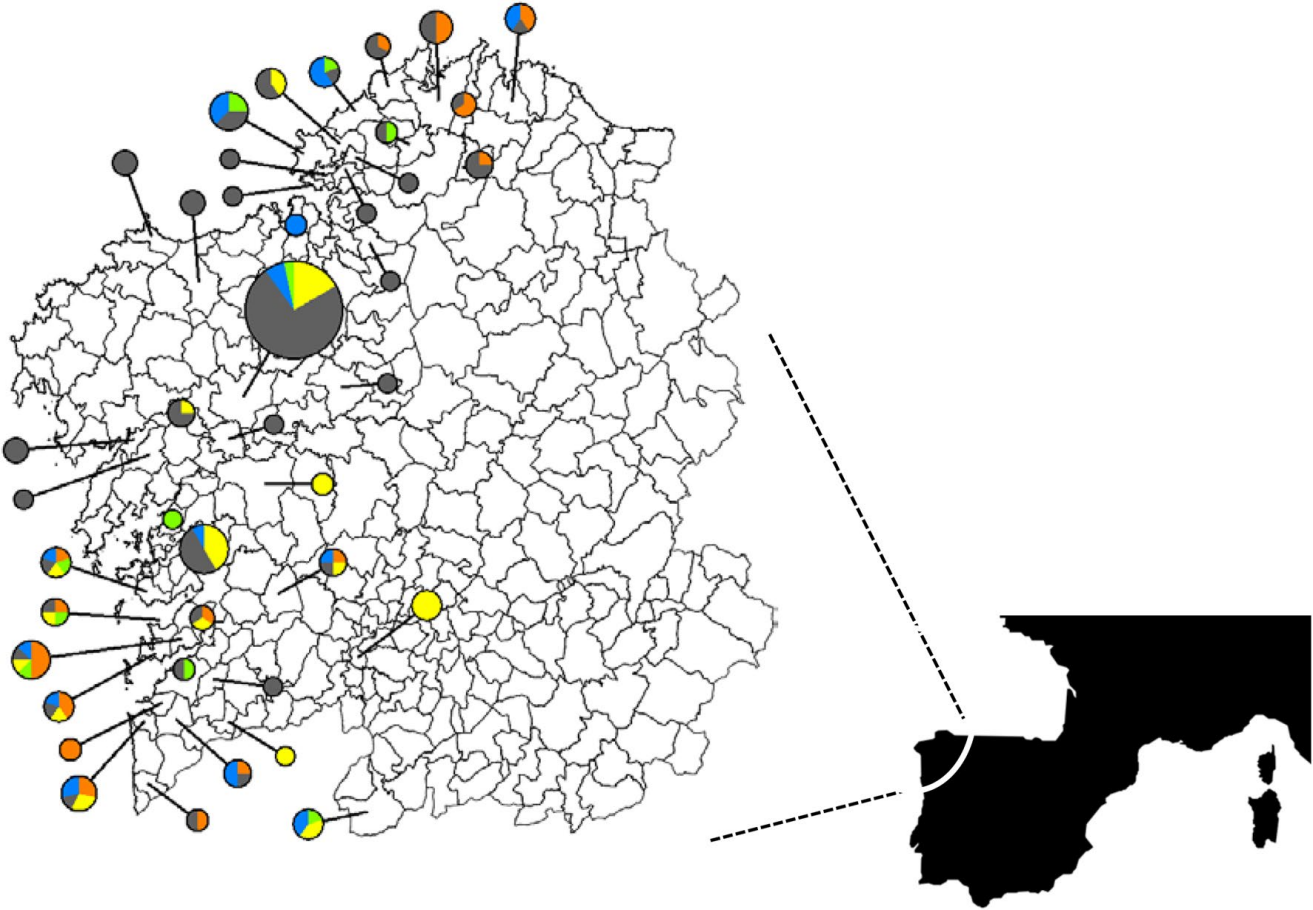
Geographic distribution of the samples. Circles identify the council of origin of the samples. Circles are drawn to scale to represent the number of samples and are color coded: *V. velutina* (grey), *V. crabro* (yellow), *Vespula* spp. (blue), *Polistes* spp. (green), *Bombus* spp. (orange). Map: South-West Europe and Galiza (enlarged).

Between August and November 2016 a total of 564 specimens of *V velutina*, *V. crabro* and other Hymenoptera belonging to genera *Bombus*, *Vespula* and *Polistes* were collected throughout the distribution area of the invasive species in Galiza (NW Iberian Peninsula) (Fig. 4). Specimens were captured using homemade traps or directly from nests (mainly *V. velutina*, *V. crabro*). Traps were filled with 80 ml of liquid bait: 95% alcohol free dark beer (Super Bock; Super Bock Group SGPS, S.A.), 4% white wine (various brands were used) and 1% grenadine syrup (Rives). Traps were fitted with a fine mesh to avoid insects from drowning in the bait and producing cross-contamination (Supplementary Figure S2). Captured insects were collected daily. Whole nests were removed by the fire brigade of Santiago de Compostela at night, when most of the hornets were inside the nest. This means that the specimens collected are a random sample of the population of the nest.

### Sample processing and DNA extraction

All captured specimens were classified into five groups: *V. velutina*, *V. crabro*, *Vespula* spp*.* (including mostly *V. germanica* and/or *V. vulgaris*), *Polistes* spp*.* (*P. austroccidentalis*, *P. dominula*, *P. nimpha*) and *Bombus* spp. (*B. terrestris*, *B. pascuorum* and other species) and preserved in 80% EtOH at 4 °C. Before dissection, each specimen was thoroughly washed in fresh 80% EtOH and rinsed three times in sterile distilled water. Abdomens were dissected and abdominal contents (including midgut, Malpighian tubules and fat body) of each specimen were extracted and homogenised separately in a 1.5 μL tube using disposable plastic pestles (VWR). Dissection tools and materials were cleaned with fresh 5% bleach for 20 min, rinsed two times in sterile distilled water, once in 80% EtOH and air-dried prior to manipulation of each specimen. A fraction of the homogenates of one to five specimens of the same species from each sampling location was combined in a pooled sample (depending on availability). In total there were 167 samples representing the populations of *V. velutina* (79), *V. crabro* (31), *Vespula* spp*.* (21)*, Polistes* spp*.* (11), and *Bombus* spp*.* (25). The number of individuals pooled in each sample was found to be unrelated to the number of parasite species or to the fraction of positive samples (Supplementary Figure S3). This probably reflects that most pooled samples were made up with individuals collected from the same nest, so there was little variation between specimens. Whole genomic DNA was extracted using the phenol–chloroform method: 100 μL of homogenate were digested overnight at 56 °C in 300 μL of lysis buffer (50 mM Tris–HCl pH 8, 100 mM EDTA, 100 mM NaCl, 1% SDS) with 5 μL of proteinase K (~ 20 mg/mL, Thermo Scientific) and DNA was extracted with phenol:chloroform:isoamyl alcohol (Sigma-Aldrich), precipitated with isopropanol, washed in 80% EtOH and resuspended in 100 μL of PCR grade water.

### Microscopic detection of Nosematids

Air-dried smears of homogenates from the pooled samples were stained with Calcofluor White (CFW) (0.1% w/v CFW stock solution diluted 3:7 in a 10% w/v KOH, 10% v/v Glycerine solution). CFW binds to the cell wall of *Nosema* spores and after being excited in UV emits in blue. Microscopic slides were observed at 400 × on a Nikon Eclipse C1000M fluorescent microscope using a UV 2-A fluorescent filter (Nikon; excitation filter: 330–380 nm, dichroic mirror: 400 nm, barrier filter: 420 nm).

### Molecular detection

The presence of parasites was determined by PCR amplification of selected marker loci and Sanger sequencing of PCR amplicons. The *small subunit* rRNA (*SSU* rRNA) loci were used for the detection of Nosematidae and Lipotrophidae species, respectively, and the DNA-directed RNA polymerase II subunit (*rpb1*) and Type II topoisomerase II (*topo II*) for Trypanosomatidae. Primer details are given in Table S6.

PCR amplifications were performed using Phusion High-Fidelity PCR Kit (Thermo Scientific) in a total volume of 15 μL under manufacturer’s conditions. DNA templates were 1:20 dilutions (in H_2_O) of the DNA pools. PCR cycling was as follows: initial denaturation at 98 °C for 30′; 35 cycles of melting at 98 °C for 10″, annealing for 30″ and extension at 72 °C for 30″; and final extension at 72 °C for 5′. Annealing temperatures are shown in Table S6.

Five microliters of PCR products were tested by electrophoresis in 2% agarose gels. When PCR yields were too low for direct sequencing, additional reamplifications for 10–15 cycles were performed under the same conditions, using 1 μL of a 1/20 dilution of the initial PCR product as templates.

### Sequencing and cloning of PCR products

PCR products were purified using NZYGelpure kit (NZYTech, Lda.) following manufacturer’s instructions and sent to GATC-Biotech (Eurofins Scientific) for Sanger sequencing of both strands. Sequences were corrected for accurate base calling by comparison of both strands using CodonCode Aligner (CodonCode Corporation). Sequence alignments were performed with ClustalW^83^ and manually edited with BioEdit^84^. Sequences have been deposited in GenBank under accession numbers: MW288771–MW288823, MW284939–MW284941 and MW284942–MW284950.

The presence of double peaks throughout some of the sequence reads were taken as evidence for genetic variation in the sample. In these cases, the PCR products were cloned using CloneJET PCR Cloning Kit (ThermoFisher Scientific Inc.) and DH5α *Escherichia coli* competent cells (Invitrogen) under manufacturer’s conditions using half of the indicated volumes. Plasmids were isolated using the NZYMiniprep Kit (NZYtech) and 5 clones per locus were sent to GATC-Biotech for Sanger sequencing.

### Species identification and phylogenetic analysis

Identification of parasite species was performed by comparing the resulting haplotypes with relevant reference sequences from each marker locus available in public databases (GenBank and ENA). For Trypanosomatidae the data from the two loci (*rpb1* and *topoII*) were taken independently: parasites detected by one marker locus were taken as true positives. For the samples with more than one parasite (mixed samples), only the species whose presence was further confirmed by sequencing the clones were considered. In a few cases the haplotypes were a chimera of fragments from more than one species. Given that the chance that they corresponded to true between-species recombinants is negligible, they were taken as experimental artefacts during PCR amplification and were excluded from the analyses.

Phylogenetic analyses were conducted using MEGA version 6^85^. The evolutionary relationships were inferred using the Neighbor-Joining (NJ) method, and evolutionary distances were computed at all sites using the Tamura 3-parameter method for *SSU-rRNA* sequences and at synonymous sites using the Nei-Gojobori method for *rpb1* and *topoII*. Bootstrap values were calculated using 500 replicates.

### Parasite diversity

To assess how parasite diversity varied across hosts we used the Shannon’s diversity index (*H*), which provides a quantitative measure of the species diversity by combining abundance and evenness of the species present in a dataset, and the Shannon’s equitability index (*E*_*H*_), which is the ratio of the *H* relative to its maximum value and measures the evenness in the distribution of species (takes values between 0 and 1). 95% CIs were estimated assuming that *H* follows a normal distribution^86^,

1 $$H = - \mathop \sum \limits_{S = 1}^{S} p_{i} \ln p_{i}$$

2 $$E_{H} = \frac{H}{\ln S}$$

where *S* and *p*_*i*_ are the number of species and the proportion of individuals of the *i*th species in each dataset, respectively.

## Supplementary Information

Supplementary Figures.

Supplementary Tables.
