# Supplementary material for: Identification of pathogens in the invasive hornet Vespa velutina and in native Hymenoptera (Apidae, Vespidae) from SW-Europe
Source: Sci Rep. 2021 May 27;11:11233. doi: 10.1038/s41598-021-90615-7 (PMC8160249; doi:10.1038/s41598-021-90615-7)
Supplement: Supplementary file 1 — Supplementary Figures. [file 41598_2021_90615_MOESM1_ESM.pptx]

## Slide 1
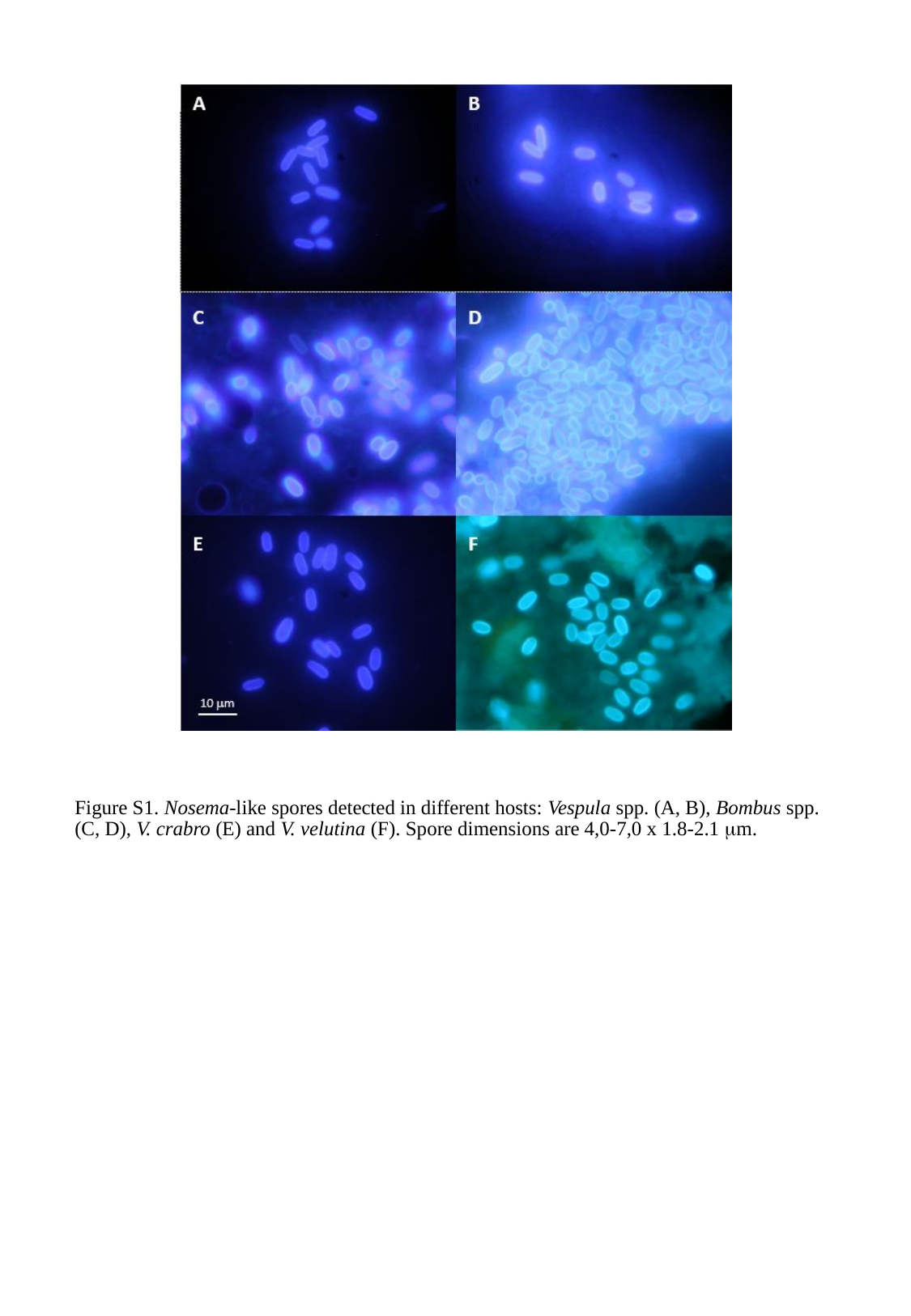

Figure S1. Nosema-like spores detected in different hosts: Vespula spp. (A, B), Bombus spp. (C, D), V. crabro (E) and V. velutina (F). Spore dimensions are 4,0-7,0 x 1.8-2.1 mm.

## Slide 2
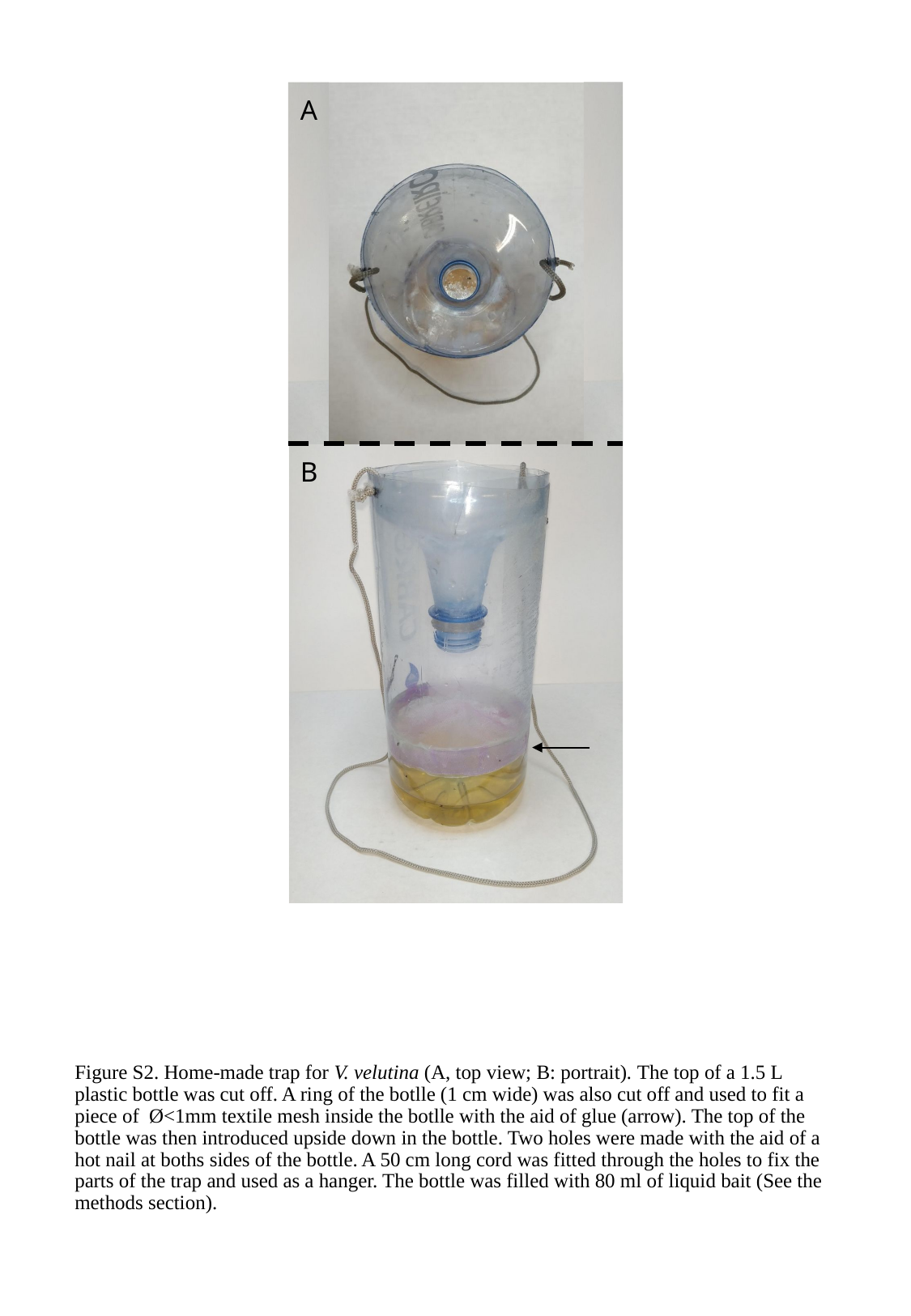

# A
B
Figure S2. Home-made trap for V. velutina (A, top view; B: portrait). The top of a 1.5 L plastic bottle was cut off. A ring of the botlle (1 cm wide) was also cut off and used to fit a piece of Ø<1mm textile mesh inside the botlle with the aid of glue (arrow). The top of the bottle was then introduced upside down in the bottle. Two holes were made with the aid of a hot nail at boths sides of the bottle. A 50 cm long cord was fitted through the holes to fix the parts of the trap and used as a hanger. The bottle was filled with 80 ml of liquid bait (See the methods section).

## Slide 3
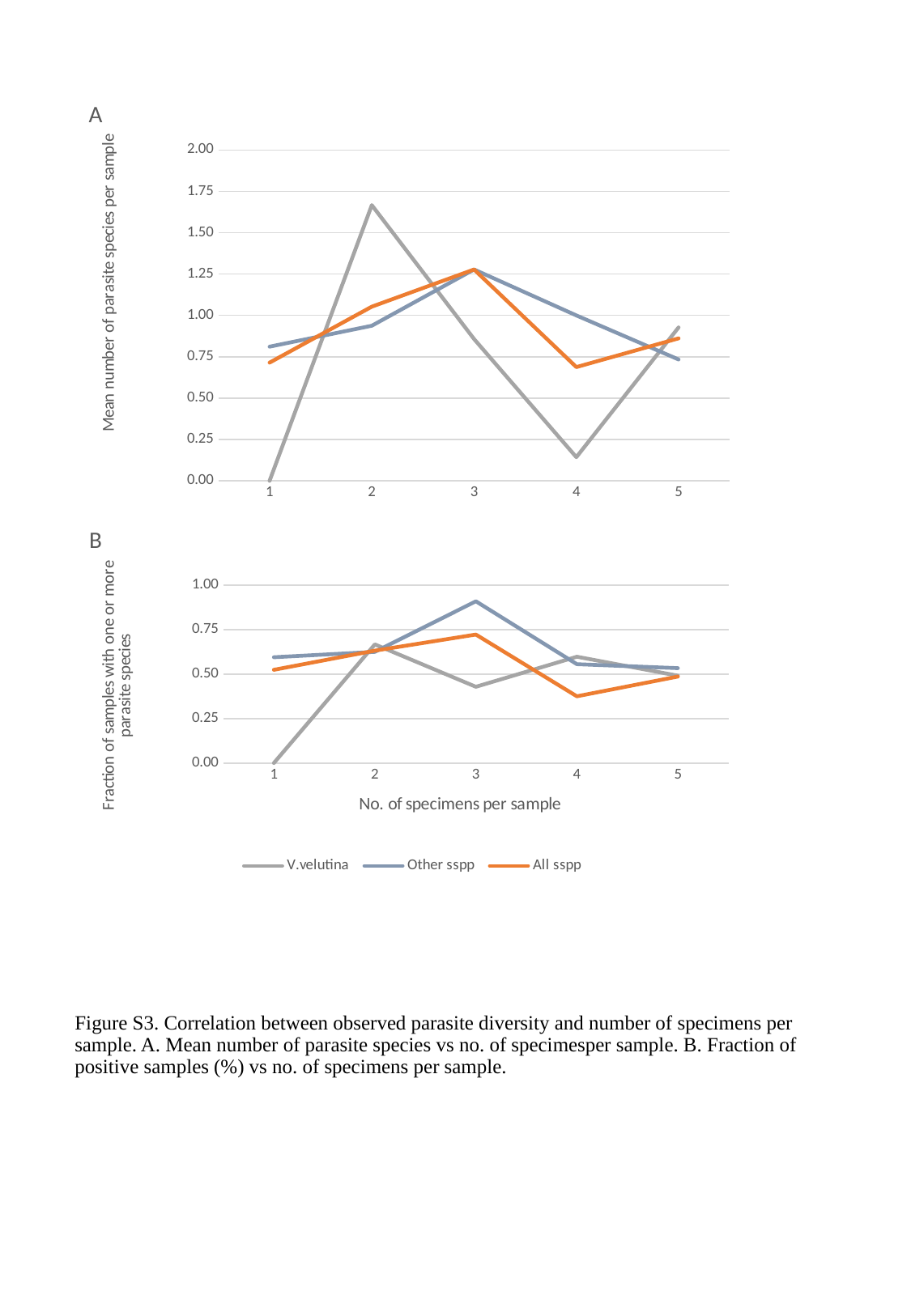

### Chart: A
| Category | V.velutina | Other sspp | All sspp |
|---|---|---|---|
### Chart: B
| Category | V.velutina | Other sspp | All sspp |
|---|---|---|---|Figure S3. Correlation between observed parasite diversity and number of specimens per sample. A. Mean number of parasite species vs no. of specimesper sample. B. Fraction of positive samples (%) vs no. of specimens per sample.
